# The Role of SR-BI in sepsis: leveraging mechanistic insights to advance precision steroid therapy

**DOI:** 10.3389/fimmu.2025.1643395

**Published:** 2025-07-28

**Authors:** Ling Guo, Qian Wang, Xiang-An Li

**Affiliations:** ^1^ Saha Cardiovascular Research Center, University of Kentucky, Lexington, KY, United States; ^2^ Lexington VA Healthcare System, Lexington, KY, United States; ^3^ Department of Physiology, University of Kentucky, Lexington, KY, United States

**Keywords:** precision medicine, sepsis, scavenger receptor BI, adrenal stress response, glucocorticoid

## Abstract

According to the Surviving Sepsis Campaign, 50.3% of septic shock patients received steroid/glucocorticoid (GC) therapy. However, whether GC therapy is beneficial and who might benefit from it are hotly debated. Initial guidelines recommended GC therapy for septic patients with adrenal insufficiency, but this has since been retracted. Recent studies using animal models of adrenal insufficiency have shed light on the mechanisms, demonstrating that the adrenal stress response is a part of the host response that is essential for control inflammatory response in sepsis and the adrenal insufficiency is a risk factor for sepsis. This perspective review explores the limitations of GC therapy through the lens of GC biology, with a particular focus on the role of scavenger receptor class B type I (SR-BI) in mediating the adrenal stress response. We highlight the mechanisms of how SR-BI-mediated adrenal stress response contributes to the regulation of hyperinflammation and innate immune responses. By integrating mechanistic insights with the limitations of GC therapy, we advocate for a precision medicine approach to GC therapy in sepsis– selectively applying GC therapy for patients with adrenal insufficiency, not without.

## Background

Sepsis is a major cause of mortality and morbidity, affecting 49 million people annually (1). It is caused a dysregulated host response to infection (2). When an infection happens, immune cells recognize the invading microorganism using pattern recognition receptors. This triggers the innate immune system, releasing cytokines, chemokines and nitric oxide, to fight infection. However, hyper activation of the host response causes organ injury, leading to organ dysfunction and death (3, 4).

Despite the significant role of inflammation in organ injury, many trials targeting inflammatory signaling have had little impact on patient survival (5). One potential limitation is that these therapies were applied nonselectively to all septic patients. In reality, septic patients have heterogeneous subtypes; some exhibit a hyperinflammatory response while other show a hypoinflammatory response. Anti-inflammation therapy may benefit those with a hyperinflammatory response but could harm those with a hypoinflammatory response. Additionally, the inflammatory response can shift from hyperinflammation to hypoinflammation depending on the stage of sepsis, requiring timely targeting of the inflammatory response (6). Given the complexity of sepsis, there is a growing call for an endotype-based precision medicine approach for sepsis therapy (7–11). In this translational review, we discuss the potential limitations of steroid/glucocorticoid (GC) therapy for sepsis regarding the target and timing of GC therapy, review the role of scavenger receptor BI (SR-BI)-mediated adrenal stress response in sepsis. By integrating mechanistic insights with the limitations of GC therapy, we advocate for a precision medicine approach to GC therapy in sepsis– selectively applying GC therapy for patients with adrenal insufficiency, not without.

## Glucocorticoid, inducible glucocorticoid and adrenal insufficiency in sepsis

GC is produced in adrenal gland and presents in circulation at 20–200 ng/ml at physiological conditions (12). GC has potent activity in regulation of inflammatory response (please refer to review article for mechanism of GC regulation of inflammatory signaling (13)). A lack of physiological levels of GC is lethal without treatment, as shown in patients with Addison’s disease and in mice undergoing adrenalectomy (14). GC acts through its receptor, glucocorticoid receptor (GR) (15). Mice lacking GR in macrophage, endothelial, dendritic, or T cells are susceptible to sepsis (16–19). These early studies clearly demonstrated critical protective roles of GC-GR signaling in sepsis.

A striking feature of GC in sepsis is its inducible nature. GC production is rapidly upregulated by 5-10-fold in response to septic stress (20). We call this inducible or induced GC (iGC) (8, 9, 21). Importantly, iGC is closely related to a common condition/phenotype in septic patients, called relative adrenal insufficiency (RAI). Patients with RAI have insufficient iGC production in response to stress, which is diagnosed by a delta total cortisol of < 9µg/dL post-ACTH stimulation (22). 25-60% of septic patients develop RAI (15, 22–24). Numerous clinical studies showed that RAI is associated with a poor prognosis of sepsis (22, 25), but some studies failed to find such correlation (26). Nevertheless, the contribution of RAI, the major type of adrenal insufficiency, to sepsis and the pathogenesis caused by RAI are largely unknown.

In 2008, the term “RAI” was replaced by “critical illness-related corticosteroid insufficiency” (CIRCI) (27). CIRCI is defined by inadequate cellular corticosteroid activity for the severity of the patient’s critical illness. It includes all types of adrenal insufficiency: absolute adrenal insufficiency, RAI and GC resistance. It is diagnosed when a seriously ill patient has very low cortisol levels (less than 10 μg/dl) or a delta cortisol < 9 g/dl upon ACTH stimulation test. Despite some disagreement, the ACTH test is commonly used to diagnose CIRCI (28).

The absolute adrenal insufficiency refers to low plasma cortisol level and is uncommon. GC resistance refers to impaired cellular GC-GR signaling. An early study by Liberty’s group reported genome-wide GC resistance in sepsis (29). In their study, mice were subjected to cecal ligation and puncture (CLP) or sham surgery, followed by dexamethasone (DEX) treatment six hours later. RNA sequencing was performed two hours post-treatment to assess gene expression changes. The authors observed no significant gene induction following DEX administration in septic mice and concluded that sepsis induces genome-wide GC resistance. However, several issues in the experimental design and interpretation of the data may undermine this conclusion. At 8 hours post-CLP, mice were under significant septic stress, and endogenous GC levels had already peaked. According to Figure S2 of RNA-seq data, 60% of genes upregulated by DEX in sham mice were also upregulated by CLP alone (227/376 genes), and 68% of genes downregulated by DEX in sham mice were similarly downregulated by CLP alone (113/165 genes). These findings show that GC/GR signaling remains active at this time point, challenging the assertion of genome-wide GC resistance. GC resistance is typically defined as impaired GR signaling despite the presence of GCs. In this context, the relevance of additional GR activation by exogenous DEX, when endogenous GC levels are already maximal, is unclear. To test the hypothesis that early GR activation leads to exhaustion, the authors performed adrenalectomy (ADX) and found that septic ADX mice failed to respond to DEX (Figure S2C). They interpreted this as evidence to support the hypothesis that early HPA axis activation causes GC resistance. However, this experiment is problematic: 90% of ADX mice died within 10 hours post-CLP as indicated in Figure S1C of the article, raising concerns about the validity of RNA-seq data collected just 2 hours before death. Moreover, DEX supplementation rescued septic ADX mice (Figure S1K), which is inconsistent with the presence of functional GR resistance. Another earlier study has also shown that GC supplementation as late as 18 hours post-CLP rescues mice with adrenal insufficiency (30). Given these inconsistencies, the data presented in the article does not convincingly support the conclusion of genome-wide GR resistance in sepsis. While GC resistance has been extensively studied in chronic diseases, it remains an underexplored area in the context of sepsis/critical illness.

As the absolute adrenal insufficiency is less common and no method to diagnose GC resistance in septic patients, we focus on RAI/CIRCI in this review.

## GC therapy for sepsis is highly controversial

GC therapy has been extensively investigated in septic patients. Early studies using high-dose GC failed to demonstrate a survival benefit (31). Given the high prevalence of relative adrenal insufficiency (RAI) in sepsis, subsequent approaches have focused on administering low-dose GC in septic shock to meet the presumed increased physiological demand (13, 15, 23, 24). The French trial reported a significant reduction in mortality among septic shock patients with RAI who received GC therapy (32). However, the CORTICUS trial, which included a less severely ill cohort, did not replicate these findings (33). Meta-analyses have attempted to reconcile these conflicting results, but conclusions remain inconclusive (34–36). The HYPRESS trial evaluated hydrocortisone in patients with sepsis without shock and found no reduction in the progression to septic shock or improvement in survival within 14 days (37). In contrast, the APROCCHSS trial, which tested a combination of hydrocortisone and fludrocortisone in patients with severe vasopressor-dependent septic shock, demonstrated a significant reduction in both 90- and 180-day mortality (38). The large-scale ADRENAL trial, involving over 3,800 patients with septic shock, did not show a survival benefit from GC therapy (39). Notably, this trial did not stratify patients based on RAI or CIRCI status. Overall, the efficacy of GC therapy in sepsis remains controversial, particularly regarding whether treatment should be tailored based on adrenal function status (40, 41). For a comprehensive overview, refer to **Table 1**, which summarizes key clinical trials of GC therapy in sepsis.

**Table 1:** Summary of literature on clinical trials of glucocorticoid (GC) therapy.

Study/source	Model/population	GC used	Key findings
Schumer et al., Ann Surg, 1976 (42)	Septic shock	dexamethasone or methylprednisolone	Corticosteroids significantly reduced mortality in septic shock.
Sprung et al., N Engl J Med, 1984 (43)	Septic shock	Methylprednisolone, dexamethasone	Corticosteroids do not improve overall survival in patients with severe septic shock, but they may be beneficial when used in the early stages or in specific patient subgroups.
Bone et al., N Engl J Med, 1987 (31)	Severe sepsis and septic shock	Methylprednisolone	High-dose corticosteroids offer no benefit in treating severe sepsis and shock.
Slotman et al., Crit Care Med, 1993 (44)	Severe sepsis and septic shock	Methylprednisolone	High-dose methylprednisolone significantly increases blood urea nitrogen and bilirubin levels in severe sepsis. Its potential adverse effects should be considered.
Bollaert et al., Crit Care Med, 1998 (45)	Septic shock	Hydrocortisone	Modest-dose hydrocortisone improves hemodynamics and survival in pressor-dependent septic shock, independent of adrenal insufficiency.
Briegel et al., Crit Care Med, 1999 (46)	Septic shock	Hydrocortisone	Stress-dose hydrocortisone shortened vasopressor duration and hastened organ recovery in septic shock.
Annane et al., Jama, 2002 (32)	Septic shock and relative adrenal insufficiency	Hydrocortisone	Seven-day low-dose hydrocortisone and fludrocortisone therapy reduced mortality in septic shock patients with relative adrenal insufficiency, without increasing adverse events.
Keh et al., Am J Respir Crit Care Med, 2003 (47)	Septic shock	Hydrocortisone	Hydrocortisone restored hemodynamic stability and promoted an anti-inflammatory, rather than immunosuppressive, response to stress.
Oppert et al., Crit Care Med, 2005 (48)	Septic shock	Hydrocortisone	Low-dose hydrocortisone accelerates shock reversal and reduces proinflammatory cytokines in early hyperdynamic septic shock, with hemodynamic effects linked to cortisol levels and immune effects independent of adrenal reserve.
Fernández et al., Hepatology, 2006 (49)	Cirrhosis and septic shock	Hydrocortisone	Relative adrenal insufficiency is common in advanced cirrhosis with septic shock, and hydrocortisone treatment is associated with frequent shock resolution and improved survival.
Annane et al., Crit Care Med, 2006 (50)	Septic shock	Hydrocortisone	7-day low-dose corticosteroids improved outcomes in septic shock patients with early ARDS who were nonresponders, but not in responders or those without ARDS.
Loisa et al., Crit Care, 2007 (51)	Septic shock	Hydrocortisone	Continuous hydrocortisone infusion in septic shock facilitates strict normoglycemia and reduces nursing workload for glucose control.
Weber-Carstens et al., Intensive Care Med, 2007 (52)	Septic shock	Hydrocortisone	Hydrocortisone bolus injections can cause variable, significant blood glucose spikes in septic shock patients, potentially leading to fluctuations. Continuous infusion is therefore preferred for better glycemic control.
Sprung et al., N Engl J Med, 2008 (33)	Septic shock and relative adrenal insufficiency	Hydrocortisone	Hydrocortisone did not improve survival or shock reversal overall in septic shock patients, including corticotropin nonresponders, although it hastened shock reversal in those who eventually recovered.
Arabi et al., Cmaj, 2010 (53)	Cirrhosis and septic shock	Hydrocortisone	In cirrhotic patients with septic shock, hydrocortisone initially improved hemodynamics, it did not reduce mortality and was linked to increased adverse effects.
Huh et al., Respirology, 2011 (54)	Septic shock	Hydrocortisone	No difference in 28-day mortality was observed between septic shock patients with relative adrenal insufficiency treated with low-dose hydrocortisone for 3 or 7 days.
Moreno et al., Intensive Care Med 2011 (55)	Septic shock	Hydrocortisone	Hydrocortisone-treated patients showed faster reduction in overall organ dysfunction, mainly due to quicker cardiovascular recovery, but this did not translate into lower mortality.
Keh et al, Jama, 2016 (37)	Severe sepsis	Hydrocortisone	In adults with severe sepsis without septic shock, hydrocortisone did not reduce the 14-day risk of septic shock, providing no support for its use in this group.
Annane et al., N Engl J Med, 2018 (38)	Septic shock	Hydrocortisone plus fludrocortisone	In septic shock patients, 90-day all-cause mortality was lower with hydrocortisone plus fludrocortisone treatment compared to placebo.
Antcliffe et al., Am J Respir Crit Care Med, 2019 (56)	Transcriptomic sepsis response signatures (SRSs)	hydrocortisone	Septic shock transcriptomic profiles predicted corticosteroid response; patients with the immunocompetent SRS2 endotype had higher mortality when treated with corticosteroids versus placebo.
Venkatesh et al., Anesthesiology, 2019 (57)	Septic shock (Sepsis-3) diagnostic criteria or APROCCHSS inclusion criteria	Hydrocortisone	In Sepsis-3 or APROCCHSS subjects, continuous hydrocortisone infusion did not reduce 90-day mortality compared to placebo in septic shock.
Moskowitz et al., Intensive Care Med, 2020 (58)	Septic shock	Combination, hydrocortisone	In septic shock patients, ascorbic acid-corticosteroid-thiamine combo did not significantly reduce SOFA scores compared to placebo, providing no support for routine use.
Meduri et al., Intensive Care Med, 2020 (59)	ARDS patients	Methylprednisolone	Early, prolonged GC therapy improves survival and reduces inflammation.
Sevransky et al., Jama, 2021 (60)	Sepsis-induced respiratory and/or cardiovascular dysfunction	Combination, hydrocortisone	In critically ill sepsis patients, vitamin C, thiamine, and hydrocortisone treatment did not significantly increase ventilator- or vasopressor-free days within 30 days; however, early trial termination may have limited detection of meaningful effects.
Wong et al., Crit Care Med, 2021 (61)	Gene expression-based endotypes of pediatric septic shock	Hydrocortisone	corticosteroid exposure may increase mortality in septic shock patients with endotype A.
Cohen et al., Intensive Care Med, 2021 (62)	Septic shock	Hydrocortisone	Adrenocortical candidate gene expression was not linked to mortality. Higher GLCCI1 predicted faster shock resolution and higher BHSD1 predicted slower resolution with hydrocortisone.
Walsham et al, Intensive Care Med, 2024 (63)	Septic shock	Hydrocortisone, enteral fludrocortisone	Enteral fludrocortisone achieved variable plasma levels in septic shock patients, reflecting inconsistent absorption; its addition to hydrocortisone did not shorten time to shock resolution.
Heming et al, Lancet Respir Med, 2024 (64)	Community acquired pneumonia (CAP) and septic shock	Hydrocortisone plus fludrocortisone	hydrocortisone plus fludrocortisone reduced mortality in septic shock patients with CAP. The analysis was underpowered to separate ARDS and CAP effects, and no mortality benefit was seen in the non-CAP subgroup.
Donaldson et al, JAMA Netw Open, 2025 (65)	Septic shock	Hydrocortisone	In septic shock patients, IV hydrocortisone was linked to a reduced risk of requiring new kidney replacement therapy.

## The Problematic diagnosis of RAI/CIRCI and its implications for GC therapy in sepsis

RAI or CIRCI is defined as “insufficient glucocorticoid (GC) relative to increased physiological demand” or “inadequate cellular corticosteroid activity for the severity of critical illness.” The ACTH stimulation test is widely used to diagnose RAI/CIRCI, but its application in septic patients remains controversial. Several experts have questioned the validity of using the ACTH test in the context of sepsis (72, 73), and the most recent clinical guidelines have not reached a consensus on its utility for diagnosing CIRCI (28). The test is designed to assess the adrenal stress response by measuring inducible GC (iGC) production stimulated by ACTH. While appropriate for non-septic patients, its interpretation in septic patients is problematic. These patients are already under extreme physiological stress and typically exhibit elevated levels of endogenous iGC. In this context, the ACTH test evaluates the incremental adrenal response—referred to as “delta iGC”—on top of an already heightened iGC. This raises critical questions: What is the physiological significance of a low delta iGC (e.g., < 9 μg/dL)? Does it truly reflect “insufficient GC relative to demand”?

To investigate the reliability of the ACTH stimulation test in sepsis, we conducted the test in a murine model of sepsis (74). Strikingly, the ACTH test identified the majority of mice as having adrenal insufficiency during the early and intermediate stages of sepsis—even those with a demonstrably intact adrenal stress response. More concerning, ACTH administration significantly elevated inflammatory cytokine levels to lethal thresholds, resulting in a moderate but measurable increase in mortality. These findings highlight critical flaws in the use of the ACTH test for diagnosing RAI/CIRCI in sepsis. Not only does the test risk misclassifying patients and misguiding GC therapy, but it may also provoke a harmful inflammatory response under septic conditions. This raises the possibility that the inconclusive outcomes of clinical trials on GC therapy may stem not from the ineffectiveness of targeting adrenal insufficiency, but from the flawed diagnostic criteria used to identify it. This underscores the urgent need for a deeper understanding of GC biology in the context of sepsis—particularly the role of the adrenal stress response (iGC production). Mechanistic studies in animal models deficient in iGC production offer a promising avenue to elucidate the precise contribution of adrenal insufficiency to sepsis pathophysiology and to refine therapeutic strategies accordingly.

## Scavenger receptor BI protects against sepsis

Scavenger receptor BI (SR-BI) is a membrane protein (75, 76). It is abundantly expressed in liver, endothelial cells and steroidogenic tissues. SR-BI functions as a high-density lipoprotein (HDL) receptor, mediating the uptake of cholesteryl ester from HDL, which is essential for reverse cholesterol transport in the liver. In SR-BI null mice, the deficiency in this receptor leads to elevated HDL levels, female infertility, autoimmune disorders when aging (77), and susceptible to atherosclerosis (78–81). Similarly, humans with loss-of-function mutations in SR-BI also exhibit impaired uptake of cholesteryl esters from HDL, elevated HDL levels, and an increased risk of coronary heart disease (82, 83). This indicates that SR-BI has similar functions in both humans and rodents [please refer to SR-BI review articles (76, 84)].

Dr. Li’s laboratory first reported SR-BI as a protective factor in sepsis (85, 86). They reported that LPS induces 90% fatality in SR-BI null mice versus 0% in wild type controls (85), and cecal ligation and puncture (CLP) induces 100% fatality in SR-BI null mice versus 20% in wild type controls (86). Using an LPS model, Dr van der Westhuyzen’s group confirmed the protective role of SR-BI in endotoxemia and showed that SR-BI null mice are susceptible to LPS-induced endotoxic death due to uncontrolled inflammation (66). They further found that SR-BI null mice lack GC production upon ACTH stimulation or LPS challenge and pretreatment of SR-BI null mice with dexamethasone 8 hours prior LPS challenge prevented the mice from LPS induced endotoxic death. Dr. Huby’s group generated adrenal specific SR-BI null (SF1CreHypoSR-BI^fl/fl^) mice and showed that the mice are more susceptible to CLP-induced septic death than control (HypoSR-BI^fl/fl^) mice (67). Using a bacterial pneumonia sepsis model (87), Gowdy et al. reported that SR-BI null mice suffer increased mortality associated with higher bacterial burden in the lung and blood, deficient in corticosterone production, higher serum cytokines, and organ injury. SR-BI null mice had significantly increased PMN recruitment and cytokine production in the infected airspace. Early efforts have revealed that SR-BI exerts its protection through multiple mechanisms including preventing nitric oxide-induced cytotoxicity (85), promoting neutrophil recruitment and LPS clearance (66, 86, 87), regulating cholesterol metabolism in liver (88) and suppressing TLR4 signaling in macrophages (86, 89, 90). These early studies establish SR-BI as a multiple protective molecule in sepsis [for detail, please refer to review article (91)].

## Scavenger receptor BI-HDL pathway is a key regulator of the adrenal stress response in sepsis

In adrenal gland, GC production is markedly induced in response to septic stress (92). In our previous study, we specifically defined iGC production as an adrenal stress response in sepsis (8). GC is derived from intracellular cholesterol. The intracellular cholesterol comes from three resources: 1) endocytosis from LDL through LDL receptor; 2) up taken from HDL through SR-BI; and 3) *de novo* synthesis. SR-BI-HDL pathway appears playing an essential role in iGC production. SR-BI null mice maintain normal basal GC levels at physiological conditions, but lack iGC under stress conditions induced by factors like LPS (66), ACTH stimulation (66), cecal ligation and puncture (CLP) (30, 67, 86), or long-term fasting (93, 94). The SR-BI null mice had normal expression in other key genes related to cholesterol *de novo* synthesis (66). Considering that rodents mainly have HDL with very low LDL in circulation, Dr. Li’s laboratory generated humanized SR-BI^-/-^ApoBtg mice (SR-BI null mice expressing ApoB) with high LDL in circulation. The mice did not produce iGC in response to ACTH stimulation or under sepsis conditions (95). Regarding the role of SR-BI in human, an early report showed that human carriers SR-BI P297S mutant, which has a 50% reduction in the uptake of cholesterol from HDL, displays a 50% reduction in iGC production to ACTH stimulation (83). In contrast, a 50% reduction in LDL receptor in familial hypercholesterolemia patients does not hinder cholesterol delivery to the adrenal cortex (96). These studies establish SR-BI-HDL pathway as a key regulator of iGC production in sepsis.

## Scavenger receptor BI-mediated adrenal stress response is an essential host response against sepsis

As discussed above, SR-BI null mice have normal basal GC levels at physiological conditions, but lack iGC under stress conditions induced by ACTH stimulation (66) or CLP (30, 67, 86). Thus, SR-BI null mice are RAI/CIRCI. Dr. Li’s laboratory generated adrenal specific SR-BI null mice by adrenal transplantation and used the mice as the first RAI animal model to determine if GC therapy benefits mice with RAI (30). They demonstrated that mice deficient in adrenal SR-BI lack iGC production in response to CLP challenge and are more susceptible to CLP-induced septic death and kidney injury. Importantly, GC treatment 2- and 18-hours post CLP effectively rescued adrenal specific SR-BI null mice. Interestingly, GC treatment caused more death in wild type, which was associated with lower plasma IL-6 levels and higher bacterial load in the blood and in the peritoneal fluid, suggesting immunosuppression in GC-treated wildtype mice.

Considering that the adrenal transplantation may disrupt catecholamine production by adrenal gland, Dr. Li’s laboratory generated adrenal specific SR-BI null (SF1CreSR-BI^fl/fl^) mice using new floxed SR-BI mice (8, 97). The SF1CreSR-BI^fl/fl^ mice were deficient in adrenal SR-BI expression but had normal SR-BI expression in other tissues. Using this new SF1CreSR-BI^fl/fl^ mice, they showed that adrenal SR-BI-specific knockout mice have impaired iGC production in response to ACTH stimulation and to CLP-induced sepsis. They demonstrated that while both wild-type and RAI mice exhibit a hyperinflammatory phenotype in the early stage of sepsis, iGC keeps the inflammatory response under control in wild-type mice. However, RAI mice experience uncontrolled hyperinflammation due to a lack of iGC. Supplementing with GC restores control of the inflammatory response in RAI mice. SF1CreSR-BI^fl/fl^ mice were susceptible to CLP-induced sepsis (6.7% survival in SF1CreSR-BI^fl/fl^ mice versus 86.4% in SRBI^fl/fl^ mice). Supplementation of a low stress dose of GC to SF1CreSR-BI^fl/fl^ mice kept the inflammatory response under control and rescued the mice. However, SR-BI^fl/fl^ mice receiving GC treatment exhibited significantly less survival compared to SR-BI^fl/fl^ mice without GC treatment.

The importance of SR-BI-mediated iGC production in pediatric sepsis was assessed in 21-day-old mice (9). Mice deficient in adrenal SR-BI were susceptible to both CLP and cecal slurry induced septic death, with survival of 88.9% in SRBI^fl/fl^ mice versus 15.4% in SF1CreSRBI^fl/fl^ mice in CLP model; 33% SRBI^fl/fl^ mice versus 0% in SF1CreSRBI^fl/fl^ mice in cecal slurry model. SF1CreSRBI^fl/fl^ mice featured persistent inflammatory responses, and were effectively rescued by administering GC 2 hours post CLP. GC treatment did not improve survival in CLP-challenged wild type mice. While GC has been shown to suppress many inflammatory signaling pathways *in vitro*, using an unbiased RNA-seq analysis, the study found that a lack of iGC production in SF1CreSRBI^fl/fl^ mice causes persistent inflammatory responses mainly due to transcriptional dysregulation of AP-1 and NF-B (9). In addition, the study found that iGC functions to control cytokine-induced secondary inflammatory response (9).

**Figure 1:**
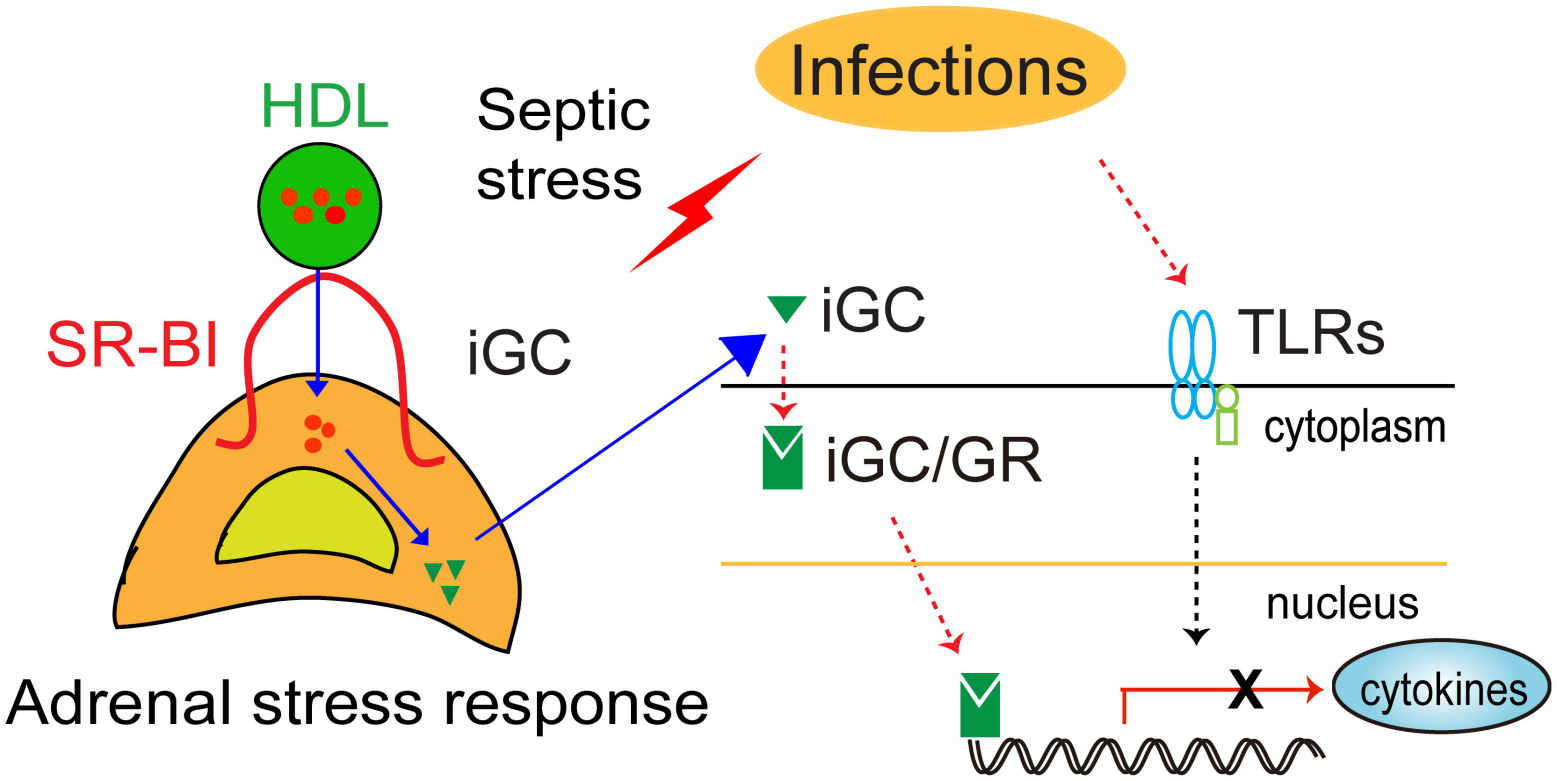
Schematic model of SR-BI-mediated adrenal stress response (iGC production) protection against sepsis. Upon infection, immune cells recognize the invading microorganism using pattern recognition receptors (TLRs). This triggers the innate immune system, releasing cytokines to fight infection. However, dysregulation of the host response causes organ injury, leading to organ dysfunction and death. In response to septic stress, adrenal SR-BI mediates the uptake of cholesterol from HDL into adrenal gland for induced glucocorticoid (iGC) production, which functions to keep the inflammatory response under control.

In sum, SR-BI mediates iGC production in sepsis. Using SR-BI null mice as a model for adrenal insufficiency, early studies demonstrated that the adrenal stress response is an essential host response, which functions to keep the inflammatory response under control. GC therapy benefits mice with adrenal insufficiency but harms mice without it (**Figure 1**).

## Translation of the mechanistic studies into a precision medicine approach to guide GC therapy for sepsis

The mechanistic findings using adrenal SR-BI null mice as a RAI model provide proof-of-concept that targeting RAI/CIRCI with GC can be an effective therapy for sepsis. However, clinical trials did not show a survival benefit of GC therapy in septic patients. There are a number of disconnections between GC therapy and the mechanisms of GC function, which may render GC therapy less effective. Let’s examine it through the lens of GC biology in sepsis.

1) Adrenal insufficiency versus diagnosis of RAI/CIRCI: RAI or CIRCI is commonly defined as “insufficient GC activity relative to increased physiological demand” or “inadequate cellular corticosteroid activity for the severity of critical illness.” However, these definitions are conceptually vague and lack mechanistic specificity, limiting their utility in guiding GC therapy. Mechanistically, sepsis triggers a robust host response, including activation of the adrenal stress axis and increased inducible glucocorticoid (iGC) production. As previously discussed, failure to mount this adrenal stress response is a recognized risk factor in sepsis, and targeting patients with impaired iGC production may improve outcomes. In clinical practice, the ACTH stimulation test is used to assess adrenal function. While appropriate for non-septic patients, its application in septic patients—who already exhibit elevated endogenous iGC due to extreme stress—raises concerns. In this context, the test measures the incremental adrenal response (delta iGC) on top of endogenous iGC. Our recent findings indicate that the ACTH test fails to elicit additional iGC production in mice with normal adrenal stress response during the early and intermediate phases of sepsis. Moreover, under septic conditions, ACTH administration can exacerbate cytokine production, potentially worsening patient outcomes (74). Consequently, the ACTH test may misclassify septic patients with an intact adrenal stress response as adrenal insufficient, leading to inappropriate GC therapy.

2) Functions of GC versus targets of GC therapy: Mechanistic studies have established that inducible glucocorticoids (iGC) play a critical role in modulating inflammation. Current clinical guidelines recommend GC therapy for septic patients experiencing shock (41), with hypotension serving as the primary criterion for intervention. **Table 2** summarizes literature on phenotype-based GC therapy in animal models. While GCs are known to support blood pressure regulation, this recommendation is largely grounded in clinical observations rather than mechanistic understanding. Hypotension in sepsis is typically a downstream consequence of organ dysfunction, which is often driven by hyperinflammation. Given that iGCs function primarily to control inflammatory responses, a mechanistically informed approach would suggest that targeting patients with iGC insufficiency and a hyperinflammatory phenotype may be more effective than relying solely on the presence of hypotension as an indicator for GC therapy. This perspective raises an important question: could a precision medicine strategy that identifies and treats patients with impaired iGC production and hyperinflammation yield better outcomes than the current one-size-fits-all approach?

**Table 2:** Summary of literature on phenotype-based glucocorticoid (GC) therapy in animal models.

Study/source	Model/population	GC used	Key findings	Precision strategy
Cai et al, J Clin Invest, 2008 (66)	SR-BI-null mice	Corticosterone	SR-BI-null mice lack inducible glucocorticoid synthesis. SR-BI is essential for the anti-inflammatory response to endotoxic shock via its roles in glucocorticoid production and LPS clearance.	Corticosterone supplementation decreased the sensitivity of SR-BI-null mice to LPS.
Gilibert et al, J Immunol, 2014 (67)	Hypo-adrenal SR-BI null mice		adrenal SR-BI is essential for HPA axis function, enabling effective glucocorticoid-mediated host defense following endotoxic shock or bacterial infection.	
KM Gowdy et al, Mucosal Immunology, 2015 (68)	SR-BI null mice (adrenal insufficiency model)	Corticosterone	SR-BI null mice showed impaired stress-induced GC production, increased mortality, and exaggerated inflammation during bacterial pneumonia. Corticosterone replacement corrected neutrophil trafficking but not mortality.	Use of genetic model to dissect adrenal contribution; GC replacement to isolate adrenal effects.
Ai et al, Crit Care Med, 2015 (69)	adrenal-specific SR-BI null mice (adrenal transplantation)	Corticosterone	Corticosteroid treatment benefits mice with adrenal insufficiency but harms mice without adrenal insufficiency.	Corticosteroids may benefit septic patients with adrenal insufficiency but harm those without, supporting the need for clinical studies to test this hypothesis.
Quatrini & Ugolini, Cellular and Molecular Immunology, 2021 (70)	Preclinical (rodent & cellular models)	Corticosterone, Dexamethasone	GC effects are highly cell- and tissue-specific; GR signaling varies by context.	Targeting GR isoforms and cell-specific delivery.
Wu et al, Front Immunol, 2022 (8)	adrenal-specific SR-BI null mice (SF1CreSR-BI^fl/fl^ mice)	Hydrocortisone	Glucocorticoid treatment benefits mice with relative adrenal insufficiency (RAI) but is harmful in mice with normal adrenal function.	Selectively applying GC therapy for a subgroup of patients with RAI.
Hobson et al., Research in Autism Spectrum Disorders, 2023 (71)	Preclinical (molecular modeling)	SEGRMs	Selective GR modulators reduce side effects while preserving efficacy.	Ligand design for selective GR activation.
Hao et al, J Infect Dis 2023 (9)	21-day-old SF1CreSR-BI^fl/fl^ mice	Hydrocortisone	Relative adrenal insufficiency mice exhibited significantly higher mortality and were effectively rescued by glucocorticoid therapy.	Use of glucocorticoids (GCs) in sepsis based on the status of relative adrenal insufficiency.

3) Timing of GC action versus timing of GC therapy: Following infection, immune effector cells rapidly initiate a robust inflammatory response characterized by the release of high levels of cytokines and chemokines. While essential for pathogen clearance, this response can become detrimental if not properly regulated, leading to tissue damage and organ dysfunction. Mechanistic studies have shown that inducible glucocorticoids (iGC) are produced early in the course of infection and play a critical role in modulating inflammation during the early and intermediate stages of sepsis. In contrast, current clinical guidelines recommend initiating GC therapy in septic patients who develop shock (41), —a condition that typically manifests in the later stages of sepsis. This temporal disconnect raises an important concern: is GC therapy being administered too late in the disease course to exert its full therapeutic benefit? If iGC’s anti-inflammatory effects are most critical during the early phases of sepsis, delayed intervention may limit the efficacy of exogenous GC therapy. This discrepancy underscores the need to re-evaluate the timing of GC administration and consider earlier, targeted intervention based on mechanistic insights.

## Conclusions - reevaluating GC therapy in sepsis through a precision medicine lens

Despite extensive clinical trials, glucocorticoid (GC) therapy has demonstrated limited impact on patient survival (5). The efficacy of GC treatment in sepsis and whether its use should be stratified based on adrenal insufficiency—remains a subject of ongoing debate (40, 41). This controversy is further complicated by the limitations of the ACTH stimulation test, which may not reliably diagnose adrenal insufficiency in septic patients.

Given the complexity and heterogeneity of sepsis, there is increasing support—including from our own studies (7–9, 21, 30)—for adopting a precision medicine approach to sepsis management (98, 99). A cornerstone of this strategy is the identification and targeted treatment of patient subgroups defined by specific endotypes (100, 101). Mechanistic studies using adrenal SR-BI knockout mice, a validated model of adrenal insufficiency, have identified adrenal insufficiency as both a risk factor and a distinct endotype in sepsis. Building on these insights, we advocate for two key shifts in clinical practice: 1) Redefining and developing improved diagnostic criteria for adrenal insufficiency in septic patients, moving beyond the limitations of current testing methods; 2) Implementing a precision medicine framework to guide GC therapy—administering treatment in a timely and selective manner to patients with confirmed adrenal insufficiency, while avoiding unnecessary use in those with an intact adrenal stress response.
